# Early and Mild Cases of Mental Disorder

**Published:** 1908-05-02

**Authors:** 


### Mental Diseases.

### EARLY AND MILD CASES OF MENTAL DISORDER.

That the mental state varies with the bodily health and vigour has been noticed from the earliest times. The severity of the variation ranges from the delirium and hallucinations accompanying specific fevers and other acute toxic conditions, to the morbid emotional feelings and wandering attention which accompany sometimes even a mild cold in the head. Bodily fatigue, too, will disturb the mental state. Shortness of breath due to running will so compel the attention to the action of the heart and lungs as to render for the time being serious thought on outside topics difficult, if not impossible. The same effect is seen in severe organic disease of the heart and lungs. Besides physical causes, there are others which are purely mental. Any emotion, even mildly felt, may bias our intellectual faculties, and if it be strong enough to break down the inhibition supplied by the intellect, will upset the mental balance. Fatigue of the higher intellectual centres from overwork or "worry," although the effects of these are noticed but little or not at all by the patient, may cause profound mental variation. The mental effect produced depends to a large extent on the idiosyncrasies of the individual. One will become delirious with quite a slight fever or intoxication, another not with a severe one: the emotion which will drive one to distraction leaves another quite unmoved.

To assign causes for an attack of mental disorder is seldom easy, and often impossible, even by one who has known the patient intimately. The earliest symptoms of mental disease are not, as a rule, recognised by the friends; indeed, the attack is often far advanced before it is realised or admitted that anything is wrong. Very frequently, when friends are pressed to give the earliest date that mental symptoms were noticed they name one, and then add, "In the light of after-events, however, we now see that before this," etc. Friends, too, are often loth to own that the trouble is mental and insist on it being a "nervous breakdown"; questions in these cases as to previous mental history are promptly scorned as displaying ignorance or a bad diagnosis. In a large number of cases all that can be said, after careful inquiry, is that such and such factors had some bearing on the ætiology. These factors, however, must be carefully sought, as the correction or removal of them will be a most important point in the treatment.

When, in practice, mental symptoms are suspected or found in a patient, three main difficulties arise: first, to make certain of the kind and extent of the symptoms; secondly, to gauge their seriousness; thirdly, to judge how best to deal with the case. These difficulties are, as a rule, greater in mild and early cases than in those of a more pronounced character.

Amongst the earliest symptoms usually noticed may be mentioned: eccentricities of conduct, change of temperament (depression or excitability), loss of interest in home or business affairs, changes in ideas of economy, impaired power of concentrating attention, alteration in emotional tendencies (especially religious), insomnia, loss of memory, and alteration of feelings towards relatives and friends. Though the appearance of any of these symptoms may not mean an attack of mental trouble, yet when any of them persist a careful examination of the mental state should be made, together with an attempt to discover the cause of the change. It must not be forgotten that the patient will often conceal his true feelings and sensations, control his temperament, and have his attention unduly stimulated whilst talking to a stranger. As a rule, however, if the examination be prolonged, the most prominent symptoms will be elicited. When the symptoms have not far developed, it may be impossible for the medical man to say for certain that the mental state is altered from the normal unless he has intimately known the patient previously; evidence of others is then essential to the correct judgment of the case.

In gauging the seriousness of the symptoms special attention should be paid to several points. Are there any signs of impulsiveness? If so, do they occur frequently, and in what form? Impulse may be mild, childish, and easy of control; or may be serious—even homicidal—and controllable by no effort on the patient's part. Suicidal tendency, however mildly displayed, demands special note. Changes in the more elementary habits—*e.g.* personal cleanliness—are of importance, showing changes in early developed centres. Refusal of food may be a serious symptom requiring special attention. Any tendency which, if not corrected, may conduce to the moral or physical harm of the patient, or those around him, must be looked on as serious.

How best to deal with an early case depends to a large extent on financial circumstances. When these permit and the symptoms are mild and harmless in character, complete change of air and scene, and removal from business, home, or other circumstances which have been present during the commencement of the attack, with a congenial companion who has good influence and control over the patient, may be all that is necessary. Quietness is usually best. Rushing round sight-seeing, travelling from place to place, and "cheerful society," are rarely to be recommended. Fresh air and occupation of body and mind is the ideal. Should the symptoms be at all serious, more supervision may be necessary. A capable mental nurse (who has been trained for mental cases) should be obtained, and the question of certification will have to be kept in view. When means are limited, the problem of early treatment is a difficult one; but it should be remembered that if the case hangs fire for long or gets worse, early certification and removal is better than late.

